# Expression of Concern: *In vivo* and *in vitro* effects of hyperplasia suppressor gene on the proliferation and apoptosis of lung adenocarcinoma A549 cells

**DOI:** 10.1042/BSR-20180391_EOC

**Published:** 2021-04-19

**Keywords:** A549 cells, Apoptosis, Gene silencing, Hyperplasia suppressor gene, Lung adenocarcinoma, Proliferation

The Editorial Office has been made aware of potential issues surrounding the scientific validity of this paper, hence has issued an expression of concern to notify readers whilst the Editorial Office investigates.

